# Effect of Essential Oils from Lemongrass and Tahiti Lime Residues on the Physicochemical Properties of Chitosan-Based Biodegradable Films

**DOI:** 10.3390/foods12091824

**Published:** 2023-04-28

**Authors:** Luis Daniel Daza, Miguel Ángel Montealegre, Angélica Sandoval Aldana, Mónica Obando, Henry Alexander Váquiro, Valeria Soledad Eim, Susana Simal

**Affiliations:** 1 Department of Chemistry, University of the Balearic Islands, Ctra Valldemossa, km 7.5, 07122 Palma de Mallorca, Spain; lddazar@ut.edu.co (L.D.D.); valeria.eim@uib.es (V.S.E.); 2 Departamento de Producción y Sanidad Vegetal, Facultad Ingeniería Agronómica, Universidad del Tolima, Ibagué 730006, Colombia; mangelmontealegre@ut.edu.co (M.Á.M.); apsandovala@ut.edu.co (A.S.A.); mobando@ut.edu.co (M.O.); havaquiro@ut.edu.co (H.A.V.)

**Keywords:** biopolymers, food packaging, biodegradable films, bio-based material

## Abstract

This work aimed to evaluate the impact of adding two essential oils (EO) from lemongrass (LEO) and Tahiti lime (TLEO) on the physical, mechanical, and thermal properties of chitosan-based biodegradable films. Six film formulations were prepared: two controls with chitosan concentrations of 1% and 1.5% *v*/*w*, two formulations combining the two chitosan concentrations with 1% LEO *v*/*v*, and two formulations combining the two chitosan concentrations with 1% TLEO *v*/*v*. The films’ morphological, water affinity, barrier, mechanical, and thermal properties were evaluated. The films’ surface showed a heterogeneous morphology without cracks, whereas the cross-section showed a porous-like structure. Adding EO to the films promoted a 35–50% decrease in crystallinity, which was associated with an increase in the elasticity (16–35%) and a decrease in the tensile strength (9.3–29.2 MPa) and Young’s modulus (190–1555 MPa) on the films. Regarding the optical properties, the opacity of the films with TLEO increased up to 500% and 439% for chitosan concentrations of 1% and 1.5%, respectively. While the increase in opacity for the films prepared with LEO was 357% and 187%, the reduction in crystallinity also reduced the resistance of the films to thermal processes, which could be explained by the reduction in the enthalpy of fusion. The thermal degradation of the films using TLEO was higher than those where LEO was used. These results were indicative of the great potential of using TLEO and LEO in biodegradable films. Likewise, this work showed an alternative for adding value to the cultivation of Tahiti lime due to the use of its residues, which is in accordance with the circular economy model. However, it was necessary to deepen the study and the use of these essential oils in the preparation of biodegradable films.

## 1. Introduction

The high demand for plastic packaging derived from petroleum has generated worldwide concern due to the environmental impact that its use can produce. In this sense, the production of biodegradable and ecological packaging becomes an alternative to reduce the impact generated by non-biodegradable packaging since they offer characteristics such as a low cost of production, bioavailability, sustainability, and biodegradability [1]. In the food industry, different materials, such as starch, gelatin, proteins, and chitosan, have been evaluated as raw materials to produce edible films and coatings [2]. Among the materials used for this purpose, chitosan has been one of the most studied due to its different properties, such as its ability to form films, antimicrobial activity, odorless quality, tastelessness, and biodegradability [3]. Other authors have reported successfully preparing biodegradable films and edible coatings from chitosan and its application as packaging in different food matrices [4,5,6]. However, chitosan-based coatings and films have some limitations that are mainly related to their mechanical properties due to high brittleness [7]. This has led researchers to seek alternatives to enhance the properties of chitosan films and coatings [8].

In addition, new consumer trends toward clean-label products and natural additives to the detriment of synthetic additives have awakened interest in active packaging with functional characteristics such as antioxidants, antibacterial, and antifungal [2]. Essential oils are secondary metabolites of plants and are primarily aromatic volatile organic compounds with biological activity [9]. These materials are highly recognized for their use in the cosmetic and medical industry due to their antiradical and antimicrobial potential. The above characteristics make essential oils (EO) an alternative to the synthetic additives used in the packaging industry [9]. Recently, different authors have evaluated the potential of chitosan films by including the EO of ginger [10], cinnamon, clove, and mustard [11], thus demonstrating the compatibility of materials and the improvement in some of their physicochemical and functional properties. However, the results observed in the literature are not conclusive, and each material and its properties depend to a large extent on the concentration and type, or source of the EO used. On the one hand, it was reported that the inclusion of orange EO, in different concentrations, to chitosan and gelatin films decreased tensile strength while increasing elongation at the break of the evaluated materials [3]. On the other hand, an increase in tensile strength was reported with a decrease in the elongation of chitosan films with the inclusion of different concentrations of cinnamon EO [12]. Therefore, there is a need to characterize and evaluate the effect of adding EO on the physicochemical properties of chitosan-based films.

The cultivation of Tahiti lime represents a productive sector of great importance worldwide, with an approximate production of 15 million tons per year [13]. However, its use and industrial exploitation generate a large volume of waste (approximately 50% of the total weight). Among the possible uses of the agro-industrial residues of Tahiti lime, the extraction of EO using different methodologies has been studied successfully [13,14]. Regarding the use of this EO as an active ingredient in the formulation of edible coatings, evaluations have been made due to its incorporation into chitosan-gelatin systems for the preservation of rainbow trout fillets [15] and in the preparation of gelatin-based coatings for shrimp life extension [16]. In both cases, an increase in the shelf life of the food matrices was observed. Despite the above, there are very few reports on the evaluation of edible films based on chitosan and Tahiti lime EO, which makes the evaluation of this type of formulation necessary.

On the other hand, the lemongrass (*Cymbopogon citratus*) EO (LEO), due to its high extraction yield and bioactive properties, has emerged as a profitable option for its use as an active principle in the preparation of biodegradable packaging with active potential [17]. Lemongrass essential oil has been successfully used as an active ingredient in the preparation of cassava starch films. Lemongrass essential oil has been successfully used as an active principle in the preparation of films. The addition of essential oil to a cassava starch film-forming solution improved the mechanical properties of the obtained films [18]. Perdana et al. [19] prepared biodegradable films based on starch (from cassava or sago) and chitosan (at different concentrations) with the inclusion of LEO and glycerol. The authors reported a reduction in the mechanical properties of the films with LEO inclusion. Additionally, the use of this coating on chilies increased the shelf life of the product by reducing weight loss and delaying ripening during storage.

Based on the availability of the Tahiti lime residues and lemongrass, as well as the high extraction yield and potential of LEO and TLEO as ingredients for the preparation of biodegradable films, this work aimed to evaluate the impact of the addition of these two EOs on the physical, mechanical, and thermal properties of chitosan-based films and compared the two ingredient systems.

## 2. Materials and Methods

### 2.1. Materials

The chitosan (Low molecular weight 50–190 kDa, Deacetylated 75–85%) was purchased from Sigma-Aldrich (Sigma Aldrich, St. Louis, MO, USA). The acetic acid (food grade) was purchased from Cimpa S.A.S. (Cimpa S.A.S., Bogotá, Colombia), and glycerol was purchased from Merck (Merck, Darmstadt, Germany). Lemongrass and Tahiti lime were purchased at a local market in the city of Ibagué (Colombia). The EOs (LEO and TLEO) were obtained from the hydrodistillation method using a Clevenger apparatus.

### 2.2. Films Preparation

Chitosan at two concentrations (1.0% and 1.5% *w*/*v*) was dissolved in 1% *v*/*v* acetic acid under agitation for 1 h at 25 °C. Then, the chitosonium acetate solution was stored at 4 °C and allowed to stand overnight.

The films were prepared using the casting method described previously by Homez-Jara et al. [5], with some modifications. Six film formulations were prepared: the chitosonium solutions at 60 °C were mixed only with glycerol (1% *w*/*v*) to prepare the control samples (Ch1 and Ch15 films); they were also mixed with the EO of lemongrass (1% *v*/*v*) (Ch1L and Ch15L films) or Tahiti lime (1% *v*/*v*) (Ch1TL and Ch15TL films). Mixing was carried out for 2 min at 10,000 rpm with a homogenizer (Ultra-Turrax T25; IKA, Staufen, Germany). Then, the solutions were vacuum filtered to remove excess bubbles. The solution (30 g) was poured into Petri dishes (diameter 8.5 cm) and dried at 40 °C for 24 h. The obtained films were stabilized for at least 24 h within a desiccator cabinet (DH-1002, Acequilab, Bogotá, Colombia) under a relative humidity (RH) of 54% (using magnesium nitrate salt) before analysis.

### 2.3. Morphology of Films

The surface and cross-sectional morphology of the films was examined using a microscope scanning electron microscopy (S–3400N, Hitachi, Düsseldorf, Germany), which operated at an accelerating voltage of 15 kV under a vacuum pressure of 40 Pa. The samples were placed on stubs over double-sided carbon tape. Then, the samples were covered with a gold layer using a rotary-pump coating system (Q150R, Quorum, Laughton, UK). The micrographs were obtained at different magnifications. For cross-sectional images, the samples were cryo-fractured under liquid nitrogen.

### 2.4. X-ray Diffraction (XRD) of Films

The X-ray diffraction patterns of the samples were obtained using a Bruker D8 ADVANCE diffractometer (Bruker, Billerica, MA, USA) with Ni-filtered Cu-Kα1 radiation (40 kV and 40 mA) and a specific wavelength of 1.5406 Å. Data collection was carried out in the 2θ range of 3.5–70° (0.02035° of step size) and at a counting time of 0.8 s/ step. Relative crystallinity was calculated using the DIFFRAC.EVA software (Bruker, Billerica, MA, USA).

### 2.5. Moisture, Swelling Power, and Solubility

The determination of the moisture content (*MC*), swelling power (*SP*), and solubility (*S*) were carried out by the gravimetric method reported by Homez-Jara et al. [5].

### 2.6. Water Vapor Permeability

Water vapor permeability (*WVP*) was determined using the ASTM method [20]. The samples were loaded into stainless steel permeability cells, where 6 mL of distilled water was deposited with an exposed area of 9.6 cm^2^. The cells were placed in a controlled chamber (25 ± 2 °C and 75 ± 2% RH), and the weight was recorded every hour for 8 h. To ensure a uniform condition, a fan was placed inside the chamber. The RH was assumed to be maintained at 100% within the cells. The *WVP* (g m^−1^ s^−1^ Pa^−1^) was calculated using Equation (1)

(1) $$WVP=aFT/tAP_{sat}({RH}_{1}-{RH}_{2})$$

where *a* is the mass of the permeability cell (g) at time *t* (s); *FT* is the film thickness (m); *A* is the exposed area (m^2^); *Psat* is the saturation vapor pressure at the test temperature (Pa); *RH*_1_ is the RH of the water contained in the cells, expressed as a fraction; and *RH*_2_ is the RH of the chamber, which can be expressed as a fraction.

### 2.7. Optical Properties

The color parameters were assessed using a Minolta colorimeter (Cr 410, Konica Minolta, Tokyo, Japan). The results were expressed as a yellow index (*YI*) (CIELAB color space). Opacity (OP) was calculated using the absorbance of the material measured at 600 nm using a spectrophotometer (Genesys 10S UV-VIS, Thermo Fisher Scientific, Waltham, MA, USA) and Equation (2):

(2) $$OP={Abs}_{600}/FT$$

where *Abs*_600_ is the absorbance at 600 nm, and *FT* is the film thickness (mm).

### 2.8. Mechanical Properties

The mechanical properties of the films were evaluated using the ASTM D882 methodology [21]. Specimens with dimensions of 5.0 cm × 1.0 cm were loaded in a texturometer (LS1, Lloyd Ltd., Largo, FL, USA) equipped with a 1 kN load cell. The initial distance between the gaps was 4 cm, and the speed of the analysis was 5 mm/min. The analysis was carried out at room temperature (~22 ± 2 °C). The tensile strength (TS, MPa), Young’s modulus (YM, MPa), and elongation at break (EB, %) were calculated using Nexygen Plus software (Lloyd Ltd., Largo, FL, USA, Version 3.0).

### 2.9. Attenuated Total Reflectance-Fourier Transform Infrared Spectroscopy (ATR-FTIR)

Fourier-transform infrared spectroscopy (ATR-FTIR) analysis was carried out using a Fourier-transform infrared spectroscopy (Tensor 27, Bruker Optics, Ettlingen, Germany) equipped with the Platinum ATR accessory (Bruker). The measurements were recorded over a range of 4000 to 500 cm^−1^, with a resolution of 4 cm^−1^ and 40 scans at room temperature (~22 ± 2 °C).

### 2.10. Thermal Analysis

Thermogravimetric analysis (TGA) was performed using a thermogravimetric analyzer (SDT 2960, TA Instruments, Tokyo, Japan). Samples (5–10 mg) were loaded into porcelain microcapsules. The samples were heated at a temperature range between 30 °C and 600 °C, with a heating rate of 20 °C, under a nitrogen stream. The samples’ weight loss (WL) and thermal degradation temperature (TD) were calculated using Universal Analysis 2000 V 4.5 A software (TA Instruments, Japan).

The differential scanning calorimetry (DSC) analysis was performed using a differential scanning calorimeter (DSC 3+, star system, Mettler Toledo, Columbus, OH, USA). The sample (approximately 5 mg) was placed into aluminum pans, which were hermetically sealed. Then, the samples were subjected to the following temperature program: cooling from room temperature to –60 °C at a rate of 20 °C min^−1^, followed by heating up to 250 °C at a rate of 20 °C min^−1^. The process was carried out in a nitrogen atmosphere using an empty pan as a reference. The melting temperature (*Tm*) and melting enthalpy (Δ*H*) of each sample were determined using Mettler-Toledo STAR^e^ software, version 14.00 (Mettler Toledo, Columbus, OH, USA).

### 2.11. Statistical Analysis

The results were reported as the mean and standard deviation of at least three replicates. An analysis of variance was used to determine significant differences (*p* < 0.05) between the samples. Multiple range tests, with the Tukey–Kramer range test, were used to determine which means were significantly different (*p* < 0.05).

## 3. Results and Discussion

### 3.1. Morphology of Films

**Figure 1:**
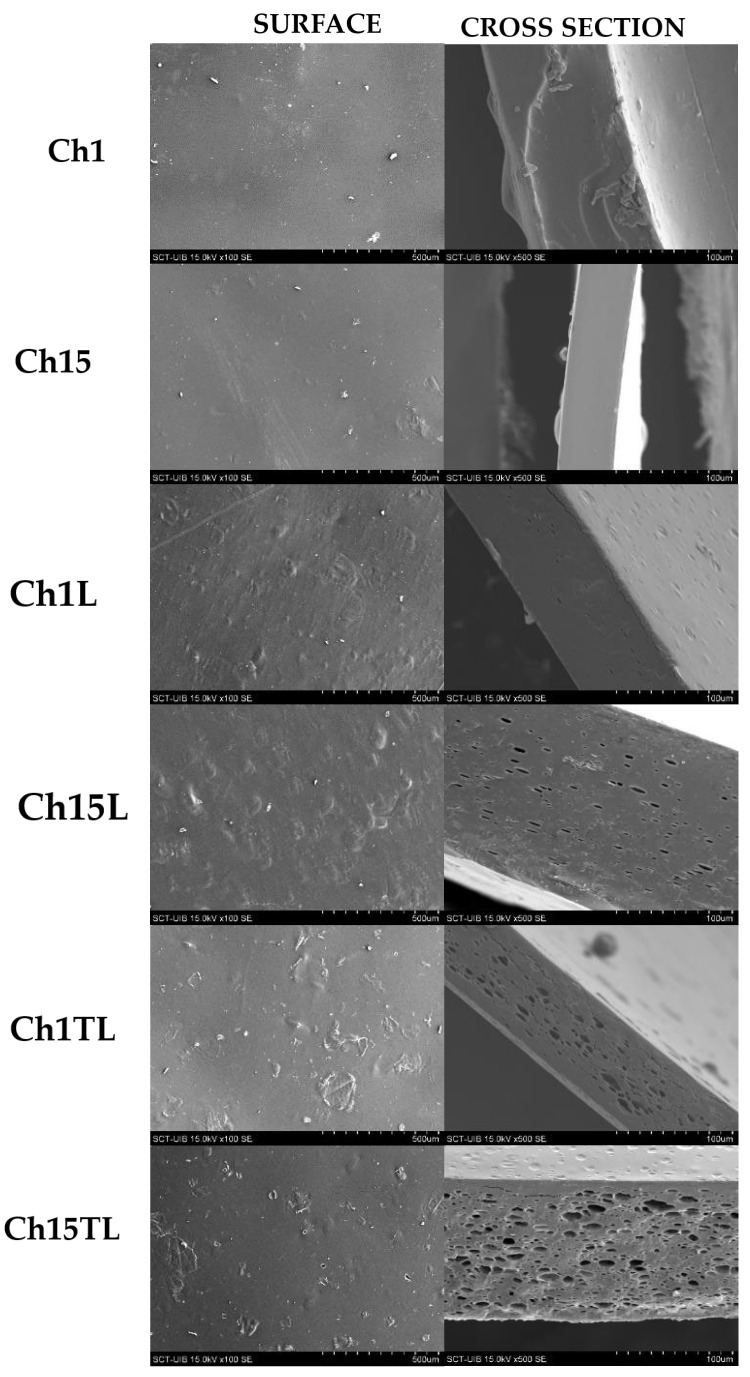
SEM micrograph of surface (×100) and cross-section (×500) of control films (Ch1 and Ch15); films with LEO (Ch1L and Ch15L); films with TLEO (Ch1TL and Ch15TL).

SEM micrographs of the film are shown in Figure 1. The control films obtained from chitosan and glycerol mixtures (Ch1 and Ch15) showed continuous, smooth surfaces with some irregularities and impurities. Likewise, the cross-section of these samples was homogeneous and without cracks. This morphology has been reported previously for films prepared with these ingredients, demonstrating high compatibility between these two materials [5]. Adding EO to the chitosan and glycerol mixture produced films with a surface without cracks but with heterogeneous morphology (bubble-like surface), indicating the partial separation of hydrophobic EO from the aqueous phase during drying. Additionally, the cross-section showed a porous-like structure, which was caused by the oil-in-water (*o*/*w*) emulsion. This phenomenon was more evident in films prepared with TLEO, which may be influenced by the chemotype of the EO or by the more significant interaction of the components of the films prepared with LEO. Similar observations on the morphology of the films prepared with chitosan, nisin, and *Perilla frutescense* EO (at 0.5%, 1.0% and 1.5% *v*/*v*) using an ultrasonic frequency and power of 40 kHz and 50 W have been previously reported [22]. The authors observed that with an increase in the EO concentration, the surface heterogeneity and porosity observed in the cross-section of the films increased. Additionally, a similar behavior was reported in chitosan films that had added lemon, cinnamon, and thyme essential oils. Although a homogeneous distribution of the EO droplets was observed in the materials, high concentrations of EO generated defects such as flocculation and coalescence, which led to an increase in pore size [23].

### 3.2. X-ray Diffraction (XRD) of Edible Films

**Figure 2:**
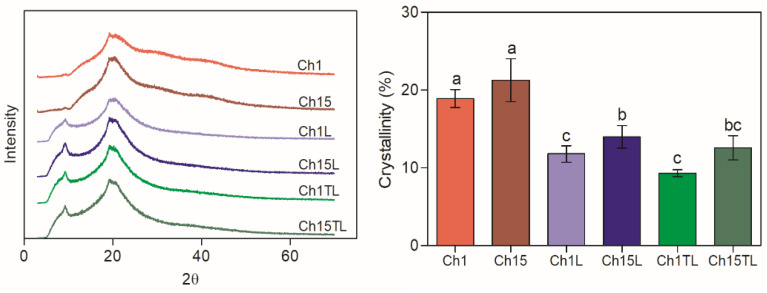
XRD diffractograms and crystallinity (%) of chitosan–essential oils films. Different letters for the same characteristic indicate significant differences among the samples (*p* < 0.05).

The X-ray diffraction patterns and relative crystallinity values of the films are shown in Figure 2. All samples showed peaks at around 9.8° and 20° 2θ (Figure 2), which were associated with the anhydrous crystalline (crystals forms I) and aqueous crystal character (crystals forms II), respectively [24]. It was observed that the intensity of the peak corresponding to crystal form I increased in the samples prepared with both EOs compared to the control samples (Ch1 and Ch15), which presented a higher intensity in the peak corresponding to crystal form II. The increase in the intensity of the type I peak in the samples prepared with EOs. This may be due to the separation and partial disorder of the chitosan chains due to the addition of some chemical groups that were present in the EOs. These results confirm those obtained for films prepared by chitosan and *Perilla frutescens* (L.) Britt. EO, in which an increase in the separation of chitosan chains could be observed with an increase in the concentration of EO added [25]. As a result of the loss of order in the chains and internal structures, a decrease in relative crystallinity could be observed. In this work, the addition of TLEO and LEO in chitosan films caused a decrease in crystallinity (Figure 2). This effect was more evident in the films prepared with the lowest chitosan concentration (1%), which could be associated with the lower ratio of chitosan: EO. The reduction in the relative crystallinity of the films can have an effect on the physical properties of the material, such as mechanical and barrier properties [19], due to the lower reorganization of the polymer chains [26].

### 3.3. Moisture Content, Swelling Power, and Solubility

**Figure 3:**
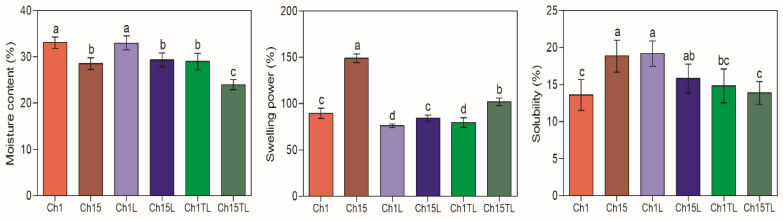
Moisture content (% wet basis), swelling power (%), and solubility (%) of chitosan–essential oil films. Means and standard deviations. Different letters for the same characteristic indicate significant differences among the samples (*p* < 0.05).

*MC*, *SP,* and *S* are properties related to the potential use of films as they can affect barrier and degradation properties [1]. The *MC* of the samples ranged between 23.9% to 33.1% (Figure 3). In all the films with a chitosan concentration of 1% (Ch1, Ch1L and Ch1TL), the *MC* was higher than the *MC* observed for films prepared with the highest chitosan concentration (Ch15, Ch15L and Ch15TL). A higher concentration of chitosan produced a more significant interaction between the components and the rearrangement of the structures due to hydrogen bonding and Van der Waals forces, which produced a more significant entrapment of the water molecules and decreased free water in the films. Similar improvements in water-holding capacity observations were made for films based on chitosan, sodium caseinate, and rosemary EO [27]. The authors attributed an increase in the film’s moisture due to the existence of free hydrophilic groups from chitosan since the main interaction of EO was with caseinate.

The *SP* of the films showed a decrease between 12% and 15% in the films prepared with 1% chitosan and LEO or TLEO, respectively, and between 33% and 44% in films prepared with 1.5% chitosan and LEO or TLEO, respectively (*p* < 0.05). *SP* has been related to the ionization of amino or carboxyl groups, the dissociation of hydrogen and ionic bonds, polymer relaxation, and crosslinking interactions between chitosan and EOs [23]. In addition, a greater *SP* was observed in samples prepared with a higher concentration of chitosan (1.5%) in both systems (LEO and TLEO). A reduction in *SP* was also observed for the inclusion of different concentrations of EO from lemon, thyme, and cinnamon in chitosan biodegradable films, which could be associated with the increase in cross-linked interactions between chitosan and the oil phase [23,28].

Regarding *S*, this parameter decreased in films prepared with the highest concentration of chitosan and EOs compared to the control. In contrast, the opposite happened for the films prepared with the lowest chitosan concentration (Figure 3). However, no statistical differences were observed between the same systems (LEO or TLEO) at any chitosan concentration. The non-specific trend of *S* may be due to heterogeneity due to the creaming or coalescence that EOs may undergo during the drying process of the films. Additionally, it was observed that the addition of TLEO generated a reduction in *S* compared to the materials, including LEO or the controls, which could be associated with greater resistance to dissolution in aqueous systems or in the presence of matrices with high moisture content. This behavior was also observed in films prepared with chitosan and with the addition of *Plectranthus amboinicus* EO. The authors observed that an increase in the concentration of the EO in films showed a tendency to decrease *S* up to a critical concentration where this parameter increased again [29].

### 3.4. Water Vapor Permeability

The *WVP* values of control films were 2.5 ± 0.3 g m^−1^ s^−1^ Pa^−1^ (Ch1 film) and 2.41 ± 0.19 g m^−1^ s^−1^ Pa^−1^ (Ch15 film), without observing significant differences (*p* > 0.05) between both samples. Similarly, adding EO to the formulation did not significantly affect the *WVP* (*p* > 0.05) of films. Despite adding a lipophilic component to the matrix that could decrease the affinity for water in the material (increase the hydrophobic nature), the barrier properties of the films depended on the sum of different factors such as crystallinity, oil distribution in the matrix, oil concentration, film homogeneity, and microstructure [27]. As observed in previous sections, the addition of LEO and TLEO increased the hydrophobic nature of the films, which led to an increase in the free space between the chitosan chains (more open structure) and a decrease in the crystallinity of the material. Thus, the net effect of adding lipophilic components to the films could be neutralized. Similar results were reported by Di Giussepe et al. [27] for films based on chitosan, sodium caseinate, and EO from rosemary. The authors concluded that the permeability of the materials evaluated did not depend solely on the addition of lipophilic components but also on their microstructure, which could be affected by the physical state of the EO and its distribution in the polymer matrix.

### 3.5. Optical Properties

**Figure 4:**
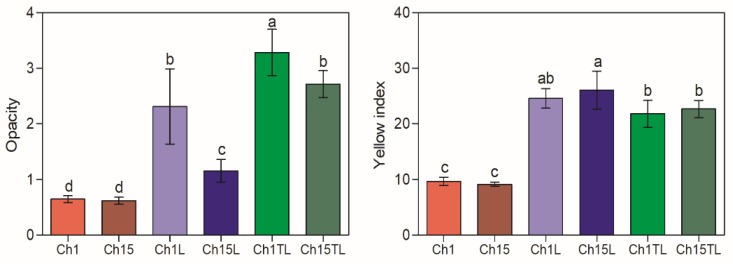
Opacity and yellow index of chitosan–essential oil films. Means and standard deviations. Different letters for the same characteristic indicate significant differences among the samples (*p* < 0.05).

Optical properties such as *OP* and *YI* are helpful when evaluating packaging materials as they determine their potential use and consumer acceptability. It was observed that the addition of either of the two EOs significantly affected the optical properties of the films (Figure 4) (*p* < 0.05). Regarding opacity, the greatest increase in this parameter was observed in the Ch1TL sample, with an increase of more than 500% for the value observed by the control, followed by Ch15TL, Ch1L, and Ch15L, with gains of 439%, 357%, and 187%, respectively. The increase in opacity of these materials could be associated with a decrease in transparency due to the emulsion formation process causing a high turbidity blocking effect in the casting solution and, therefore, in the film [30]. This reduction in transparency with the addition of EOs has been previously reported for films prepared with chitosan and EOs of *Carum copticum* (340% opacity increase) [30], clove (119% opacity increase) [31], and rosemary (112% opacity increase) [27]. Although food packaging with low transparency can be associated with the presence of defects or with consumer rejection due to a negative evaluation of products, this type of packaging presents benefits such as barriers against UV-Vis light [31]. The packaging that offers this type of barrier represents an alternative that can be used in products susceptible to lipids and protein oxidation [32].

The tendency to form films with a yellowish coloration is characteristic of samples prepared with chitosan and can be associated with β-1-4 linked 2-amino-2-deoxy-D-glucopyranose repeated units in its structure [33,34]. Similar to opacity behavior, the *YI* values of the analyzed samples significantly increased with the addition of EO (Figure 4). As expected, the greatest increase in *YI* was observed in samples prepared with EO and the largest chitosan concentration. The rise in *YI* could be associated with the color of EOs, the interactions of these with chitosan, and the higher opacity of the films. The values obtained for the control samples agreed with those reported for films prepared with pure chitosan [35]. However, they presented lower values than those reported by Jahed et al. [30] for chitosan films, including 5% *Carum copticum* EO, but similar to those observed for films with the inclusion of nail EO at concentrations below 1% [31].

### 3.6. Mechanical Properties

**Figure 5:**
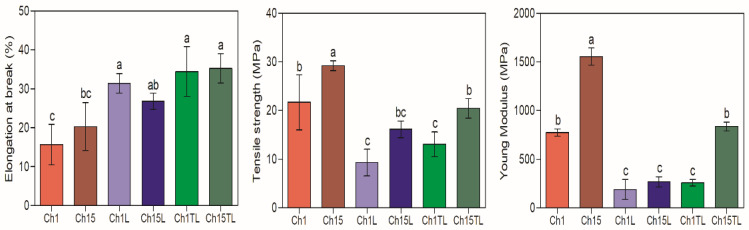
Elongation at break (%), tensile strength (MPa) and Young’s modulus (MPa) of chitosan–essential oil films. Means and standard deviations. Different letters for the same characteristic indicate significant differences among the samples (*p* < 0.05).

The results obtained in the determination of *EB*, *TS* and *YM* are shown in Figure 5. The addition of EOs (LEO and TLEO) in the formulation improved the *EB* of tested samples compared to the control films by up to 200% (*p* < 0.05). Films prepared with LEO and TLEO showed better *EB* results than the control samples (Figure 5). The increase in *EB* with the inclusion of EOs was also observed in chitosan films prepared with the inclusion of D-limonene and orange EOs, with increases of up to 230% and 167% for films with the highest EO content (1%; *w*/*w* in the case of D-limonene and *v*/*v* in the case of orange oil), respectively [3,36]. This suggests that adding EOs and forming an emulsion produces deformable droplets that can penetrate the film’s matrix and increase the free space between the chitosan chains, thus increasing flexibility. Likewise, variable changes have been reported in the mechanical properties of films prepared with chitosan and the different EOs associated with these materials’ chemical composition and source [23]. Peng and Li. [16] prepared chitosan films, which were added with lemon, thyme and cinnamon EOs, and observed an increase in TS in the films with lemon oil, while they observed a reduction in films with the other two types of EOs.

In contrast to *EB*, the addition of EOs to the formulation generated a decrease in the values for *TS* and *YM*. In the case of *TS*, the greatest reduction was observed in the samples obtained with LEO, with a decrease of 57% (1% chitosan samples) and 45% (1.5% chitosan samples) for the value of the control samples (*p* < 0.05). At the same time, the samples with TLEO showed a reduction in *TS* by 40% (1% chitosan samples) and 30% (1.5% chitosan samples) (*p* < 0.05). Regarding the *YM*, a significant decrease between 46% and 83% for the value of the control samples was observed (*p* < 0.05) (Figure 5). A reduction in these parameters was expected because they were associated with greater rigidity in the films. Therefore, a material with a higher elasticity could generally display a lower *YM* value. This inverse relationship between elasticity with *TS* and *YM* has been previously reported for chitosan films enriched with the EO of lemon, thyme, cinnamon, and orange [3,12,23]. Thus, adding EO could reduce the interaction force between the polymer chains and, therefore, reduce their rigidity, which leads to a decrease in *YM*.

### 3.7. Attenuated Total Reflectance-Fourier Transform Infrared Spectroscopy (ATR-FTIR)

**Figure 6:**
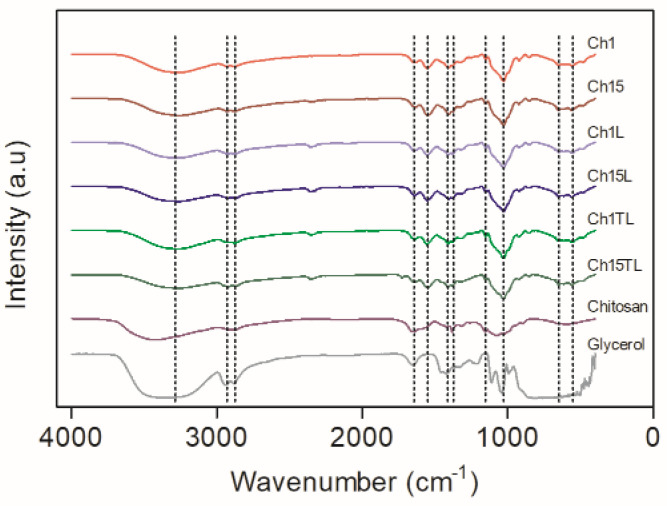
FTIR spectrums of chitosan–essential oil films.

ATR-FTIR was used to evaluate the possible interaction between materials in the formulation of chitosan-based biodegradable films. Figure 6 shows the spectra obtained for the samples analyzed. All samples presented a broad band between 3000 and 3700 cm^−1^ (for films, the band is between 3000 cm^−1^ and 3600 cm^−1^), which is related to the stretching of intra and intermolecular hydroxyl groups (–O–H) and –CH_2_–OH stretching vibrations [30]. This band also can be related to the –N–H stretching mode of chitosan, indicating an overlapping of these two vibration modes [5]. The bands observed at 2930 and 2870 cm^−1^ could be assigned to C–H bonds’ asymmetric and symmetric stretching modes [29,35]. While the bands observed at 1640 cm^−1^ and 1555 cm^−1^ could be related to C=O stretching vibrations (amide I) and the N–H bending vibration mode (amide II), respectively. Absorption bands at 1405 cm^−1^ and 1374 cm^−1^ are related to C–N, N–H stretching modes, whereas bands at 1153 cm^−1^ and 1023 cm^−1^ correspond to the absorption of C–O–C groups [5,30]. The intermolecular interaction between chitosan and glycerol in the films can be evidenced because the inclusion of the latter generated the intensification of absorption bands in the range of 600–750 cm^−1^ corresponding to the bending vibration of hydroxyl groups. In addition, it produces a sharpening of the band, which can be related to the stretching vibration of the OH group (3000 cm^−1^ to 3700 cm^−1^) and a shift in the band corresponding to the C–O stretching vibration from 1083 cm^−1^ in pure chitosan to 1023 cm^−1^ in the samples evaluated. These observations agree with the report for chitosan films and the inclusion of *Carum copticum* EO [30]. The same authors also reported that the shift in the NH bending absorption peak of pure chitosan (1670 cm^−1^) toward a shorter wavelength in the films might be due to the interaction between chitosan and EO, which could be observed in the films, including LEO and TLEO. The latter also confirmed what was observed in chitosan films, including *Ruta graveolens* EO [37].

### 3.8. Thermal Analysis

**Figure 7:**
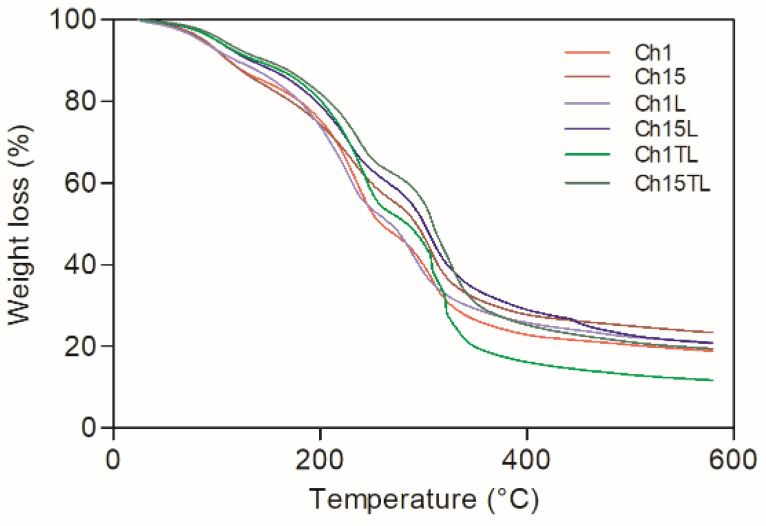
Weight loss curves of chitosan–essential oil films.

**Figure 8:**
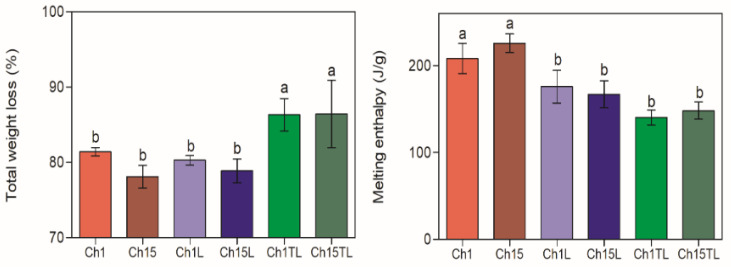
Total weight loss (%) due to thermal decomposition and melting enthalpy (J/g) of chitosan–essential oil films. Means and standard deviations. Different letters for the same characteristic indicate significant differences among the samples (*p* < 0.05).

The weight loss curves of chitosan–EO films are shown in Figure 7, and the total weight loss calculated from these curves is represented in Figure 8. The total weight loss due to thermal decomposition for the analyzed samples was from 78.1% to 86.3% (Figure 8). The decomposition took place in three stages for the control films (Ch1 and Ch15), which could be associated with the evaporation of absorbed water and residual acetic acid (49–116 °C) [30], the decomposition of glycerol (166–246 °C) [5], and the depolymerization of chitosan structures with the decomposition of functional groups such as hydroxyl and amine groups [22,33]. A significant increase in the total degradation was observed only in samples of TLEO inclusion (Ch1TL and Ch15TL) (*p* < 0.05). However, the inclusion of LEO did not significantly affect the total decomposition of films Ch1L and Ch15L.

The samples with EO added did not present significant changes in degradation stages I and II. However, the addition of TLEO in films Ch1TL and Ch15TL generated a degradation in two stages of decomposition stage III; this may be due to the interaction between EO and the chitosan chains since the latter could act as an encapsulating agent for the oil droplets once they had degraded, been released and allowed for the degradation of EO. While in the case of the samples added with LEO, a weak peak appeared above 422 °C (more evident in Ch15L than in Ch1L), which could have been related to the presence of aromatic residues from the EO. This same behavior was observed in chitosan films added with *Perilla frutescense* EO, and the intensity of the degradation peak could be associated with the EO concentration [22,25]. The results obtained in this work are consistent with those reported for chitosan films and *Carum copticum* EO [30].

An endothermic peak corresponding to the melting of the samples was observed in all the samples. No significant differences were observed among samples regarding the melting temperature, and the average figure considering the samples was 124.0 ± 6.3 °C. Thus, including EOs in the preparation of films did not generate a significant shift in *Tm.* The estimated melting enthalpy (Δ*H*) of the films is also shown in Figure 8. Despite the above, it can be observed that the films with added EO showed a decrease in the Δ*H* value, which can be associated with the fact that the inclusion of EO in the samples generated a reduction in the degree of order, and therefore, in the degree of crystallinity (see previous section). Thus, the higher the crystallinity value, the greater the resistance to temperature the samples have [38]. These results are in agreement with the results obtained for chitosan films dried at different temperatures [5]. However, there are differences with those obtained for chitosan films by including ginger EO at concentrations of 0.1, 0.2, and 0.3% (*v*/*w*) [38]. Those differences could be due to the concentrations used to prepare the materials.

## 4. Conclusions

The physicochemical properties of biodegradable films based on a low molecular weight chitosan were affected by including EOs from lemongrass (LEO) and Tahiti lime (TLEO) in the formulation. However, these EO’s had a neutral effect on the solubility and barrier properties. On the other hand, a reduction in the swelling power and crystallinity was observed, the latter associated with the loss of ordering of the polymer chains, which directly impacted the mechanical properties reducing the tensile strength and Young’s modulus but also increasing the elasticity of the material. However, a marked effect was not observed in relation to the concentration of chitosan in the evaluated system. Considering the high brittleness of the films prepared only with chitosan, the increase in their elasticity is a desired characteristic for their use in the food industry. Additionally, the inclusion of LEO and TLEO demonstrated an increase in both the opacity and the yellow index. Regarding opacity, the inclusion of TLEO presented a greater increase in this parameter compared to films prepared with LEO. Although these characteristics can be associated with negative consumer perception, they could be beneficial when preventing the damage or alterations of foods that are sensitive to photooxidation. Regarding the thermal properties, it was observed that the energy necessary for the casting of the films decreased with the inclusion of essential oils, which could be related to the loss of an organized structure or crystallinity. On the other hand, the addition of TLEO increased the total weight loss of the films, which may be associated with higher degradability. The use of TLEO as an active component in chitosan films demonstrated the potential of this EO and provided an alternative to the use of waste obtained from this material, which is in line with the circular economy trend. In general, the use of LEO and TLEO in the preparation of biodegradable films based on chitosan showed great potential. However, an optimization study is necessary to determine the best concentrations of both EOs, as well as the best concentration of chitosan for the preparation of biodegradable films.

## Data Availability

Data is contained within the article.
